# Targeting CD44 as a novel therapeutic approach for treating pancreatic cancer recurrence

**DOI:** 10.18632/oncoscience.172

**Published:** 2015-06-15

**Authors:** Maria Inés Molejon, Juan Ignacio Tellechea, Vincent Moutardier, Mohamed Gasmi, Mehdi Ouaissi, Olivier Turrini, Jean-Robert Delpero, Nelson Dusetti, Juan Iovanna

**Affiliations:** ^1^ Centre de Recherche en Cancérologie de Marseille (CRCM), INSERM U1068, CNRS UMR 7258, Aix-Marseille Université and Institut Paoli-Calmettes, Parc Scientifique et echnologique de Luminy, Marseille, France; ^2^ Hôpital Nord, Département de Chirurgie, Marseille, France; ^3^ Hôpital Nord, Département de Gastroentérologie, Marseille, France; ^4^ Hôpital de la Timone, Département de Chirurgie, Marseille, France; ^5^ Institut Paoli-Calmettes, Marseille, France

**Keywords:** pancreatic cancer, CD44, tumor recurrence, molecular targeting, cancer stem cells

## Abstract

Pancreatic ductal adenocarcinoma (PDAC) is an extraordinarily lethal disease, which, despite a more or less efficient chemotherapeutic treatment, systematically displays a rapid and uncontrolled progression towards a fatal recurrence. Determining which cells give rise to such tumor recurrence is thus crucial before an improved therapeutics outcome can be envisaged for patients with PDAC. In this regard, we recently reported that following a standard chemotherapy for PDAC, a heterogeneous subpopulation of CD44+ cells proliferates and is responsible for tumor recurrence, as shown by almost all recurrent tumor cells becoming CD44+. We designed a strategy to eliminate these cells based on a weekly administration of an anti-CD44 monoclonal antibody to human PDAC-derived xenografts in mice. We demonstrate that xenografts, which were unresponsive to gemcitabine treatment, are however sensitive to this strategy. In conclusion, CD44 represents an efficient therapeutic target in patients with recurrent PDAC.

## INTRODUCTION

### Pancreatic ductal adenocarcinoma

Pancreatic ductal adenocarcinoma (PDAC) is the most common form of pancreatic cancer and, with a 5-year survival rate of less than 5% after diagnosis and a median survival of around 6 months [1], it is also one of the deadliest cancers worldwide. The high mortality of these patients is partly due to more than three-quarters of the cases being diagnosed at too late a stage to allow surgical resection, which is the only curative option. Non-operated patients are treated with standard chemotherapies such as single drug gemcitabine [2] or with the Folfirinox protocol comprising a combination of irinotecan, 5-fluouracil and oxaliplatin [3]. However, in contrast to the steady increase in survival for most cancers over the last 15 years, advances have been very humble for patients with PDAC [4] despite the approval from the Food and Drugs Administration (FDA) of numerous promising therapies. The causes for the lack of treatment response are thought to involve the rapid progression of the disease and also the resistance shown by PDAC tumor cells against these therapies. In addition, some cancer cells became progressively resistant to drugs during the course of treatment, ultimately resulting in a non-responsive tumor to chemotherapies. Moreover, it has also been proposed that a subpopulation of cells existing in the tumor correspond to cancer stem cells (CSCs) that may be involved in cell resistance to the anticancer drugs. By definition, a CSC corresponds to a cell within a tumor that is able to self-renew and to produce the heterogeneous lineages of cancer cells that comprise the tumor [5]. Current chemotherapies clearly target proliferating cells, which mostly comprise differentiated cells, but are met with strong levels of resistance from CSCs in many tumor types [6–8] via an as yet unclear mechanism.

### Cancer stem cells

The expression of several cell-surface antigens as markers has proven useful to distinguish CSCs from normal adult tissue stem cells. Some appear to show specificity for each cancer type, while others have been described as common markers in various types of cancer. This dichotomy has led to uncertainties with regards defining true CSC markers. The existence of CSCs was first discovered in acute myelogenous leukemia [9, 10]. Al-Hajj et al., [11] then described the existence of a subpopulation expressing CD44+/CD24−/ESA+ (ephithelial-specific antigen), which were capable of inducing tumor formation in breast cancer in immunosuppressed mice. These studies allowed the identification of CSCs in several tumors, including pancreatic cancer.

One such study elegantly described the presence of CSCs in pancreatic cancer (PCSC) [5] and distinguished a subpopulation of PDAC cells expressing the phenotype CD44+/CD24+/ESA+ that were highly proliferative with high tumorigenic potential by comparison with the counterpart subpopulation expressing the phenotype CD44+/CD24−/ESA+ [5]. These triple positive cells were able to form a tumor that strongly resembled the original tumors from patients and shared all the phenotypic characteristics of a human PDAC after implantation in immunosuppressed mice [5]. Concomitantly, other authors identified a cell-type expressingthe CD133 marker at their surface and which they defined as tumorigenic and resistant to chemotherapy [8]. The function of this membrane glycoprotein is still unknown, though it is believed to confer CSC properties in various cancers [12–14]. Although CXCR4 is not a marker of tumorigenic cells, it and CD133 have been proposed to act as metastasis indicators. Indeed, silencing CD133-CXCR4 markers prevented metastasis of PDAC tumor in xenografts, leading Hermann and colleagues to proposing the CXCR4 as a target, along with CD133, to prevent metastasis [8]. An additional CSC marker has been defined as one of 17 enzyme aldehyde dehydrogenase 1 (ALDH1) isoforms, named ALDH1A1, which is a detoxifying enzyme responsible for oxidizing aldehydes to carboxylic acids and converting retinol to retinoic acid. Detection of the active ALDH1A1 using the AldeFluor assay permitted the identification of several tumor-initiating cell-enriched populations in multiple cancers such as breast, colon, pancreatic, lung, liver and ovarian cancer [15]. Recent studies revealed an association between a higher activity of intracellular ALDH1A1 and cancer cell stemness, particularly in pancreatic cancer [16, 17]. This apparent ambiguity leads to wondering whether only one type of CSC exists in pancreatic tumors or whether there are many subpopulations each expressing different markers though all displaying the capacity to form PDAC tumors.

### CD44 surface marker in PDAC

Despite the existence of many studies concerned with the identification PCSC, no specific population has yet been defined. Moreover, our recent findings suggest that there exists no combination of CSC markers able to exclusively identify a specific population of PCSC. In fact, only CD44 surface marker positive cells presented a higher expression in recurrent PDAC that were shown to be non replicative before therapy. Cells containing this marker also express, independently, CD133 and EpCAM. Naturally, many authors investigated whether cells concomitantly expressing different markers showed higher tumorigenicity. Aiming to resolve this unclear issue in PDAC, we recently studied whether cells expressing a higher number of CSC putative markers were less prone to respond to chemotherapeutic agents. We performed the quantification of CSC markers on cells derived from 14 PDACs and compared their expression, alone or together, with the IC_50_ to the 5 most frequently used drugs for treating patients with PDAC. Notably, we found no correlation between the sensitivity of the PDX-derived cells to each drug and the amount of CSC markers expressed, individually or in combination, suggesting that a higher number of CSC putative markers in a tumor does not increase its resistance to conventional PDAC treatments. Surprisingly, we found a wide variation of CSC putative markers between each pancreatic tumor [18].

**Figure 1:**
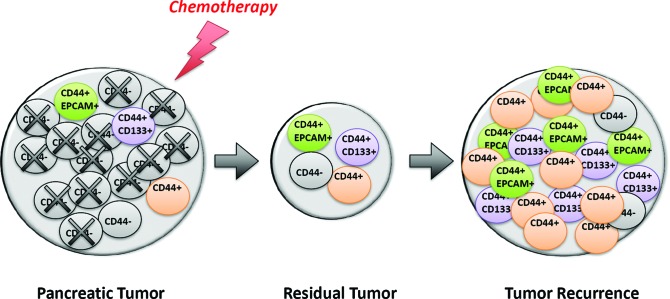
Schematic representation of the enrichment of cells expressing CD44+, CD44+/EpCAM+ or CD44+/CD133+ detected in recurrent tumor after chemotherapy Administration of anti-tumoral drugs induces an increase in the number of CD44+ cells, and these cells included expression of CSC-putative markers. More importantly, CD44+ cells acquired proliferation features after chemotherapy, demonstrating an important role of these cells as responsible of tumor recurrence.

Another important finding we recently made concerned the significant increase in the number of cells expressing CSC-associated markers in the residual tumor, following *in vivo* treatment of the PDAC xenografts with anticancer drugs; positive cells remained however a minority among all PDAC cells [18]. These data suggest that although CSCs may participate in the recurrence of PDAC they are probably not the main source of cancer cells. Defining the cellular source of residual tumor is an important point to address since these cells are responsible for the tumor recurrence, which kills so many patients with PDAC. In recent work, we defined the pattern of CSC markers in PDAC residual tumors after chemotherapies and found no discriminating differences between resistant and sensitive tumors. Also and more important, we found that treating human PDAC-derived xenografts with the most common therapies in current use for treating patients suffering a PDAC resulted in a strong enrichment of the CD44+ cell population. In some PDX submitted to the anticancer treatment, almost all the tumor cells became CD44+ (Figure 1). Interestingly, in samples from patients treated with anticancer drugs, we observed a similar CD44+ cell enrichment. Remarkably, this cell population was able to activate the cell cycle after gemcitabine therapy. Importantly, CD44+ cells comprise a heterogeneous population that includes variable numbers of CD133+ or EpCAM+ cells, suggesting that CD44+ cells encompass all the CSCs and therefore represent a very promising therapeutic candidate. The fact that a first line anticancer therapeutic agent induces a substantial increase in CD44+ cells in PDAC, leads us to hypothesize that this marker could constitute an excellent target for use in recurrent disease.

### Therapeutic targeting of CD44

Considering that the particular resistance to chemotherapies singularly incriminates CSCs, new pharmacological approaches should be developed that target these cells as specifically as possible. Although many studies have defined the existence of these putative CSCs within PDAC tumors, to our knowledge no clinical trials are underway on the use of CSC-putative markers in patients with PDAC. Defining a specific population of pancreatic CSCs on which to target therapeutic interventions appears, at least at present, impossible. Yet the CD44+ cell population includes diverse CSC-associated markers and is enriched upon PDAC recurrence. Newly designed therapeutic strategies could therefore involve the elimination of these CD44+ cells, which we have described as being responsible for tumor recurrence following anticancer treatment.

Regarding this hypothesis, we tested whether targeting CD44 could serve as a therapeutic approach for PDAC treatment using a PDX-based preclinical strategy. We showed that intraperitoneal injection of a monoclonal antibody directed against CD44 into nude mice carrying human pancreatic PDX previously treated with gemcitabine, resulted in tumor eradication. Li and collaborators [19] made similar observations in xenografted PDAC cells treated first with radiotherapy. In addition to the results in mice carrying human PDAC, we were able to demonstrate the increase in CD44+ cells in residual tumors from human patients, who had already been subjected to chemotherapy treatment. Most importantly, the development of a human anti-CD44 mAb has already been approved by the FDA (RG7356) for the treatment of hematological malignant diseases [20]. Together, these congruent data support the use of this strategy as a possible treatment for PDAC. These promising results highlight CD44 as a putative target for treating PDAC, particularly in recurrent disease. Similar results have also been reported in other cancer types such as myeloid leukemia [21] or hepatocellular carcinoma [22]. While much work is needed to fully understand the cancer machinery and CD44 regulation, this study promises an important improvement in PDAC therapeutics and subsequent improvement of patient overall outcome.

## CONCLUSIONS

Identification of new molecular targets is urgently needed in order to improve the therapeutic outcome of patients with PDAC, particularly with regards the recurrent disease. While targeting CSCs seems to be effective, a full understanding of the biology of these cells is first required in order to develop optimal therapies. Our recent data is in line with the CSC paradigm. Following a first line therapy, at the time at which PDAC became resistant, CD44+ cells not only persist in the recurrent tumor but they acquire the capacity to proliferate, suggesting an important role in tumor recurrence and thus in the particularly bad prognosis. Our results demonstrate, for the first time, that targeting CD44 surface antigen after gemcitabine treatment in human-PDAC derived xenografts, is able to eliminate the remaining tumor cells *in vivo*. More preclinical studies are now needed to confirm these results and to evaluate the toxicity of this therapeutic approach, and in parallel, to study the signaling pathway controlled by CD44 during tumor recurrence with a view to developing specific inhibitors.
